# Attention-aided hybrid transformer network for oral squamous cell carcinoma classification using histopathology images

**DOI:** 10.3389/frai.2026.1751520

**Published:** 2026-04-13

**Authors:** Meena Jagathesh, Sathiya Narayanan

**Affiliations:** School of Electronics Engineering, Vellore Institute of Technology, Chennai, India

**Keywords:** convolutional block attention mechanism, deep learning, histopathological images, oral cancer, OSCC classification, vision transformer

## Abstract

**Introduction:**

In recent years, oral cancer has become one of the most common malignant tumors. Early diagnosis of oral cancer from histopathological images will certainly reduce the severity of the disease and bring down the death rate. Several Deep Learning algorithms are available in the literature, ranging from Convolutional Neural Network models to Vision Transformer (ViT) models, to classify normal tissues and those with Oral Squamous Cell Carcinoma.

**Methods:**

This study proposes a Convolutional Block Attention-aided Transformer Network (CBA-TransNet) that combines ResNet50 with ViT. The ResNet50 acts as a backbone for extracting local features through convolutional layers, while ViT captures global context and long-range dependencies through self-attention mechanism from histopathological images. To further enhance the extracted features, the Convolutional Block Attention Mechanism (CBAM) is applied after the Feed-Forward Network layer in the ViT encoder block. The CBAM has channel and spatial attention, which helps the transformer to focus more effectively on the relevant regions of images.

**Results:**

For experiments, a publicly accessible dataset of 5192 histopathological images are used. Experimental results and analysis show that the proposed hybrid model resulted in an accuracy of 98.97%, while comparing with the pre-trained ResNet50 baseline, ViT, CNN and state-of-the-art approaches.

**Discussion:**

Experimental outcomes show that the proposed CBA-TransNet is flexible in combining both convolutional and transformer based architectures along with attention mechanisms like CBAM to extracts both local and global features. This hybrid architecture allows the model to concentrate on diagnostically significant areas, resulting in better classification.

## 1 Introduction

Cancer is a foreboding global threat which claimed the lives of almost 10 million people, representing one out of every six deaths, thus making it a scourge of modern societies (WHO, 2018). According to World Cancer Research Fund (n.d.) reports, 389,846 new oral cancer cases were documented worldwide in 2022, ranking as the 16th most common cancer globally, 12th among men, and 18th among women. Oral cancer, a global health concern, predominantly is caused by Oral Squamous Cell Carcinoma (OSCC) with high mortality, recurrence, and metastasis rates, yet approximately 50% of survival rate for locally advanced cases (Stage III and Stage IV without distant metastasis) (Bellantoni et al., 2023). It is the most prevalent cancerous tumor and is often associated with precancerized epithelium (Scully and Bedi, 2000) which includes a range of oral potentially malignant disorders such as leukoplakia, oral submucous fibrosis, oral lichen planus, erythroleukoplakia and verrucous hyperplasia. These lesions differ in the possibility of malignant transformation, with erythroleukoplakia having a greater risk than homogeneous leukoplakia, highlighting the need for prompt diagnosis and intervention. The survival chances and outcomes significantly enhance if OSCC is diagnosed and treated promptly, making thorough and precise detection vital, with biopsy being the gold standard for diagnosis (Murray Walker et al., 2003).

In recent years, technological development known as Artificial Intelligence (AI) imitates human cognitive capacities and an accomplishment that has captured scientist attention all throughout the world (Xu et al., 2025). AI has the potential to address current problems with detection and prognosis prediction in the treatment of malignant growths. By using this technology, it is easier to detect, treat and manage patients with oral cancer by reducing physician fatigue, complex data, and workload (Vinay et al., 2025). Machine Learning (ML) (Javaid et al., 2022; Rahman et al., 2023) a subdivision of AI, is revolutionizing clinical disease diagnosis by addressing the increasing complexity of healthcare. Its rapid processing of data surpasses human effectiveness, enabling quicker decision-making. ML algorithms are scalable, allowing real-time processing of large data sets from various sources. They focus on extracting useful information from available data, not necessarily from supervised sources. Deep learning (DL) (Alom et al., 2019) a subset of ML, that utilizes neural networks has become one of the leading approaches in the field of computer vision and image processing. DL algorithms have garnered a lot of attention lately due to their apparent advantages and applicability in cancer prediction especially for OSCC (Warin and Suebnukarn, 2024). The reason behind this is that it is made to help physicians make informed choices, so improving and promoting improved patient care.

Convolutional Neural Network (CNN) are good at image classification (Krizhevsky et al., 2017) they require large dataset and significant computational resources, but they are limited in addressing global contextual information and this issue is addressed by using attention-based architecture such as Vision Transformer (ViT) (Dosovitskiy et al., 2021) which uses self-attention mechanisms to capture long-range dependencies across image patches. ViT excels at global context, however, on smaller datasets, the lack of built-in priors about local shape or shift-invariance affects accuracy, which CNNs already manage well. To overcome this limitation, a hybrid architecture termed as Convolutional Block Attention-aided Transformer Network (CBA-TransNet) is proposed. It combines ResNet50 and ViT to exploit both local features and global dependencies (Li et al., 2022). Likewise, attention blocks such as the Convolutional Block Attention Module (CBAM) have been incorporated to enhance the neural networks by refining feature maps through channel and spatial attention (Woo et al., 2018). Integrating CBAM after the Feed Forward Network (FFN) layer of the transformer encoder helps the model to focus on the most informative features, thereby improving the classification performance.

This manuscript makes the following contributions.

1. The proposed CBA-TransNet uses an integration model of ResNet50 as a feature extractor with ViT to combine both local and global feature representation of OSCC images.
2. This work uses CBAM within the transformer encoder block after the FFN layer to enhance the channel and spatial attention for refining the most important features.
3. Using a publicly available dataset of 5,192 histopathology images for binary image classification, the performance of the proposed CBA-TransNet is investigated, and it was found to perform with 98.97% accuracy. Experimental results show that the proposed network outperforms ViT, CNN, Pre-trained ResNet50 and the state-of-the-art models.

The remaining sections of the paper are organized as follows: Section 2 reviews the current medical literature on the classification of oral cancer. Section 3 describes the transformer network and the classification strategies used in the proposed work. Section 4 presents the details of the proposed CBA-TransNet. Section 5 presents the experimental results and related discussions. Section 6 concludes the proposed work along with directions for further research.

## 2 Related works

Several DL and ML based techniques have been introduced in the literature recently for the diagnosis of OSCC through the analysis of medical images. ML methods use supervised learning to provide rapid and accurate evaluation of histopathology image analysis. In (Rahman et al., 2020) the classification of images is carried out in the benchmark dataset which contains OSCC database, where size based feature extraction plays an important role and the original images were hand cropped and preprocessed through color channeling and the segmentation is carried with the combination of Otsu method with the morphological operation to separate nucleus from the background, morphological and textural features are extracted. The classification of images between normal and malignant is carried out by Decision Tree (DT), Support Vector Machine (SVM), K-Nearest Neighbor (KNN), Discriminate Analysis (DA) and Logistic Regression (LR), DT performed well with 99.78%. Similarly, in Bakare et al. (2021) they used a median filter for preprocessing the images to remove background noise, the unique temporal feature extraction method is used in the preprocessed images and in the classification phase, SVM and KNN techniques were used to classify the normal and the OSCC images. Among these two methods, SVM outperformed KNN with classification accuracy of 98%. Likewise, in Rahman et al. (2018) they utilized histogram and gray scale co-occurrence matrix for textural feature extraction and the binary classification of OSCC histopathology images, is carried out using Linear SVM Classifier and they achieved 100% accuracy on 400x magnification images.

Radiomics-based texture analysis using traditional Magnetic Resonance Imaging (MRI) sequences such as T2-weighted imaging and contrast enhanced T1-weighted imaging which provides useful information regarding the tumor’s size, location and boundaries, in Ren et al. (2020) they applied Logistic Regression (LR), Random Forest (RF), and Artificial Neural Networks (ANN) for classifying texture analysis and class imbalance using Synthetic Minority Over-Sampling Technique (SMOTE). RF’s predictive performance was superior, achieving 86.3% accuracy and Receiver Operating Characteristic (ROC) curve area of 0.936. These studies demonstrate the value of traditional ML in small-data environments, they often suffer due to lack of generalizability, data imbalance and interpretability because of reliance on hand-crafted features.

By exploiting its ability to extract complex information from medical images automatically, DL has repeatedly outperformed traditional ML methods in the diagnosis and classification of oral cancer. In Welikala et al. (2020) both ResNet101 and Faster Region-Convolutional Neural Network (Faster R-CNN) achieved F1 scores of 87.07 and 41.18%, respectively. Using hyperspectral images, Jeyaraj and Nadar (2019) developed a regression-based approach and use a segmental deep CNN for classification and yielded a high accuracy of 94.5%. EfficientNetB0 outperformed the other 16 pre-trained DL models in Soni et al. (2024) which also used EfficientNetB0 in conjunction with Dual Attention Mechanism (DAN) to detect OSCC using histopathology images. The accuracy of this approach was 91.1%. In Das et al. (2023) employs a 10-layered CNN model for categorization of OSCC images and compares it with other deep learning models and attained a cross-validation accuracy of 97.86%. Transfer learning based approach using AlexNet is implemented in Rahman et al. (2022) for the classification of OSCC histopathological images and achieved a classification accuracy of 90.06%.

Different DL approaches have also been studied apart from CNNs. Capsule Networks which use dynamic routing to maintain spatial hierarchies and pose information have been used on OSCC histopathological images and achieved an accuracy of 97.35% while being more robust to rotation and affine variances (Panigrahi et al., 2022). Also, Graph Neural Networks (GNN) which take into account the spatial arrangement of the nuclei or tissue regions have been applied with CNNs to model cell to cell interactions within oral mucosal tissue. A recent study showed that CNN–GNN pipelines achieved F1-scores around 0.90 with basement membrane topology prediction, outperforming traditional CNNs in explainability and achieved an accuracy of 88.16% (Nair et al., 2022). Although these techniques are currently limited, they offer substantial promise in resolving the gaps in interpretability and structural cognition in the interpretation of OSCC images.

The reliability of classification has been improved using ensemble models. A 2D empirical wavelet transform was developed by Deo et al. (2024a) for the feature extraction and ensemble of two pre-trained models including ResNet50 and DenseNet201 for the classification of OSCC in histopathological images, and they were able to reach an accuracy of 92%. The Ensemble Classifier for Oral Malignancy Detection (ECOMD) model achieved an accuracy of 97.88% by tuning and merging the results of XceptionNet and InceptionResNetV2 (Das et al., 2024). While ensemble models enhance the stability of predictions, they often lead to higher computational costs and complications during the training and deployment of the model. Furthermore, interpretability concerns still persist unless visual explanation tools are integrated explicitly.

Hybrid models highlight how DL models can be successfully integrated with classical ML models to improve classification robustness (Al Duhayyim et al., 2023). Uses the Sailfish Optimization with Fusion based Classification (CADOC-SFOFC) algorithm. They used the fusion-based method with VGGNet-16 and ResNet models for feature extraction, and they used the Extreme Learning Machine (ELM) algorithm to classify OSCC and normal images. They used the Sailfish Optimization Algorithm (SFO) to adjust the parameters of this model, and they were able to achieve an accuracy of 98.11%. Similarly, in Ahmad et al. (2023) five pre-trained models, including Inception, Xception, InceptionResNetV2, DenseNet201, and NASNetLarge are utilized to extract features, and SVM is employed as a classifier, resulting in an accuracy of 97%. In Asnake et al. (2025) bilateral filtering and color normalization were used to preprocess histopathological images, and pre-trained models such as InceptionV3, MobileNetv3, and AlexNet were used to extract features. These features were then classified using the XGBoost classifier, demonstrating the effectiveness of combining CNN-based feature extraction with tree-based ensemble classifiers, compared with all three combinations, AlexNet with XGBoost attained better accuracy with 99%. Although in Fati et al. (2022) DL models such as AlexNet and ResNet18 were used for feature extraction and combined with the color, texture and shape features extracted from Discrete Wavelet Transform (DWT), Local Binary Pattern (LBP), Fuzzy Color Histogram (FCH), Gray-level Co-occurrence Matrix (GLCM) and classified using ANN, in which ResNet18 as a feature extractor with ANN works best with 99.3% accuracy. Hybrid pipelines improve robustness, but they suffer from end-to-end optimization due to the separation of feature extraction and classification, which is especially troublesome when integrating them into healthcare systems.

Even though CNNs are quite accurate and can automatically extract features, they may sometimes fail to recognize crucial global dependencies, and they cannot generally emphasize clinically significant regions, which affect the interpretability of the results. This limitation has led to the development of transformer-based models. ViT employs self-attention mechanisms which excel in global correlations by analyzing intricate global structures. They can extract characteristics for many small objects in an image, making them highly proficient in tasks requiring complex global structures analysis. In the study (Deo et al., 2024b), they used ViT-14 architecture for binary classification of OSCC using histopathological images and attained a classification accuracy of 97.78% by capturing both local and global contextual information within the images. Similarly, in Flügge et al. (2023) they employed the Swin Transformer architecture for OSCC detection in clinical images. It incorporates a shifted window-based self-attention mechanism to capture both local and global features, achieving an accuracy of 98.6% and an AUC of 0.99. The performance of the ViT and Swim transformer models with two CNN models, VGG19 and ResNet50, for binary classification tasks is compared using mobile-based images in (Song et al., 2024). The accuracy of the Swim transformer model was 88.7, 2.3% higher than that of the ViT model, and both transformer models perform better than the CNN models.

Although traditional ViT is capable of extracting global features using self-attention mechanisms, they completely disregard inductive biases such as locality and translation equivariance which are very important for image analysis, particularly in a medical imaging task like OSCC detection. These limitations are solved by hybrid transformer models that incorporate CNN or hand-crafted feature extractors with the transformer structures. In Li (2025) they used Cross-Attention Vision Transformer (Cross-ViT) using dual branch architecture, where image patches are divided into two different sizes and combined with features extracted by LBP, FCH, and GLCM and classified using ANN. Cross-ViT with dual branch architecture achieved the highest accuracy of 99.59% when compared with features extracted by LBP, FCH, and GLCM and integrated with Cross-ViT features. In the study (Yadav et al., 2025) they proposed a Lightweight Explainable Network (LWENet) which integrates the lightweight convolutional layers for feature extraction with the Label-Guided Attention (LGA) to ensure the label consistency and Axial Multi-Head Attention (AMHA) based ViT encoder for providing global attention to the spatial features, by implementing this technique the model achieved a precision of 96.97% and F1-score of 98.90% on Mouth and Oral Disease (MOD) dataset and a precision of 99.48% and F1-score of 98.23% on Oral Cancer Images (OCI) dataset. Table 1 shows the summary of machine learning, deep learning, transformer and other deep learning approaches for oral cancer classification.

**Table 1:** Survey on oral cancer classification techniques.

Year	Method	Dataset	Image	Technique	Accuracy
Rahman et al. (2020)	DT classifier	Private	Histopathology images	ML	99.78%
Bakare et al. (2021)	SVM	Public	Histopathology images	ML	98%
Rahman et al. (2018)	Linear SVM classifier	Private	Histopathology images	ML	100%
Ren et al. (2020)	RF with SMOTE	Private	MRI images	ML	86.3%
Welikala et al. (2020)	ResNet101	Private	Photographic images	DL	F1 score 87.07%
Jeyaraj and Nadar (2019)	Regression-based partitioned deep CNN	Private	Hyperspectral image	DL	94.5%
Soni et al. (2024)	EfficientNetB0 with DAN	Public	Histopathology images	DL	91.1%
Das et al. (2023)	10-layered CNN	Public	Histopathology images	DL	97.86%
Panigrahi et al. (2022)	Capsnet	Public	Histopathology images	Capsule Network	97.35%
Nair et al. (2022)	EfficientNet with GraphSAGE	Private	Histopathology images	Hybrid (CNN + GNN)	88.16%
Deo et al. (2024a)	Ensemble of Resnet50 and Densenet201	Private	Histopathology images	Ensemble DL	92%
Das et al. (2024)	(ECOMD) Ensemble of XceptionNet and InceptionResNetV2	Public	Histopathology images	Ensemble DL	97.88%
Al Duhayyim et al. (2023)	CADOC-SFOFC Algorithm	Public	Mobile based images	Hybrid (DL and ML)	98.11%
Ahmad et al. (2023)	Xception, Inception, InceptionResNetV2, NasNetLarge, DenseNet201 as feature extractor and SVM as classifier	Public	Histopathology images	Hybrid (DL and ML)	97%
Asnake et al. (2025)	AlexNet with XGBoost	Public	Histopathology images	Hybrid (DL and ML)	99%
Fati et al. (2022)	ResNet18 and ANN with SVM	Public	Histopathology images	Hybrid (DL and ML)	99.1%
Deo et al. (2024b)	ViT-14	Public	Histopathology images	Transformer	97.78%
Flügge et al. (2023)	Swin-Transformer	Private	Clinical photographic images	Transformer	98.6%
Song et al. (2024)	Swin-Transformer	Private	Mobile based images	Transformer	88.7%
Li (2025)	Cross-ViT	Public	Histopathology images	Transformer	99.59%
Yadav et al. (2025)	LWENet	Public	Clinical photographic images	Transformer	Precision-99.48%, F1 score-98.23%

## 3 Transformer network and classification strategies

This section provides the detailed explanation of the basic ViT model which includes transformer encoder and multi head self-attention, this section describes ResNet50 and CBAM which highlights channel and spatial attention for refinement of features.

### 3.1 Vision transformer

The input images are separated into patches through the patch embedding layer and all the patches are flattened into a one-dimensional vector. Each vectors projected into a latent space by linear projection layer, to maintain consistent dimensions across the encoder layers. Position embeddings are added to patch embeddings a learnable class token is added to the sequence of patch embeddings to collect information during attention.

#### 3.1.1 Transformer encoder block

It consists of multiple layers and each layer uses MHSA to extract relationships among patches and then uses a FFN to transform features. There is Layer Normalization (LN) and also residual connections around MHSA and FFN blocks to enhance the gradient flow. The computations for each layer are mentioned in Equations 1, 2.


$$X_{l}^{\prime }=\text{MHSA}(\mathrm{LN}(X_{l-1}))+X_{l-1}\;l=1,2\ldots L$$ (1)

$$X_{L}=\mathrm{FFN}(\mathrm{LN}(X_{l}^{\prime }))+X_{l}^{\prime }\;l=1,2\ldots L$$ (2)

Where $X_{l-1}$ denotes the output from the previous encoder layer, $X_{l}^{\prime }$ is the output after MHSA layer and the residual connection, $X_{L}$ is the final output from the *L*th encoder layer. Normalization is performed after the encoder output and then the outputs are sent to a Multi-Layer Preceptron (MLP) head for classification.

#### 3.1.2 Multi-headed self-attention

The MHSA mechanism further enhances the model’s capability by using multiple attention heads to learn diverse features. Each head focuses on different aspects of the image, helping the model to identify intricate relationships among patches. Three embeddings serve as the foundation for this technique termed as the Value (V), Key (K), and Query (Q). It is described in Equations 3–5 as follows:


Query(Q)=X×Wq (3)

Key(K)=X×Wk (4)

Value(V)=X×Wv (5)

Patch features are projected using 𝑊𝑞, 𝑊𝑘, and 𝑊𝑣 onto Q, K, and V embeddings, respectively. Self attention scores are attention weights calculated for each feature and the weight of the value embeddings is determined by this score.


$$\text{Attention}(Q,K,V)=\text{Softmax}(\frac{{\mathrm{QK}}^{T}}{\sqrt{d_{k}}})V$$ (6)

The Equation 6 indicates the calculation of attention scores. The attention scores computed using dot product for Q and K which represents patch similarity. The scaling factor stabilizes the softmax scores. These scores are converted into probabilities which allow the model to focus on the most relevant patches. The extracted features are utilized for image classification in the MLP head that generates the class probabilities for OSCC or Normal classification.

### 3.2 ResNet50

ResNet50 is a deep CNN which comprises of 50 layers as in (He et al., 2016), it uses skip connections to solve the issue of vanishing gradients and the training of deep networks is completed efficiently. It consists of a stack of convolutions, batch normalization followed by ReLU activations, assembled into bottleneck residual modules that enhance the representational capability of the network while maintaining computational efficiency. In this method the classification head of the pre-trained ResNet50 is removed and only the convolutional layers are retained to extract the feature maps. The pre-trained weights help the models to benefits the improved feature generalization on smaller and specialized medical image datasets. The extracted feature maps from pre-trained ResNet50 are forwarded to the ViT layers.

### 3.3 Convolutional block attention mechanism (CBAM)

**Figure 1:**
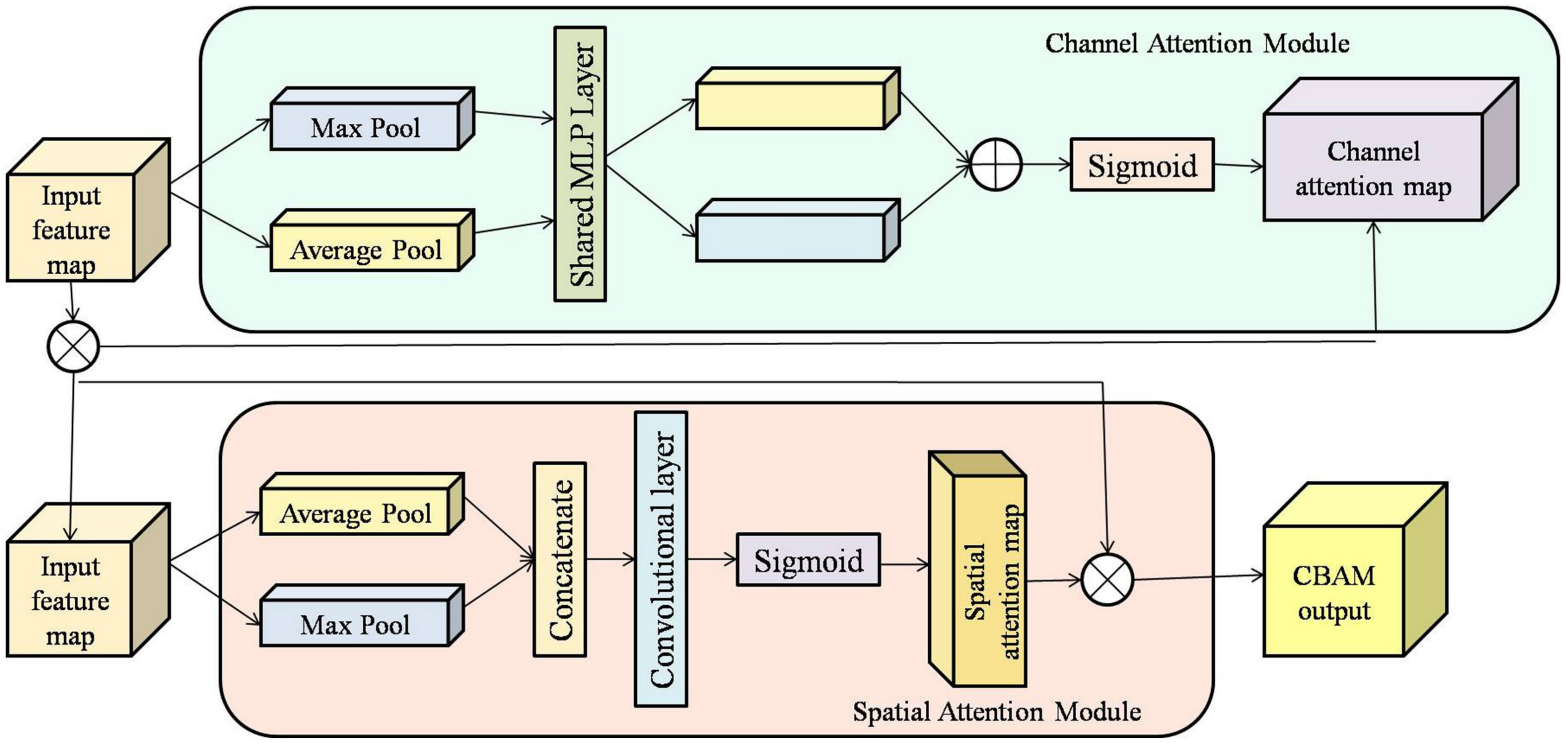
Block diagram of CBAM.

The CBAM mirrors the workings of the human brain by utilizing a lightweight yet powerful technique to heighten the significance of features in a neural network. As shown in Figure 1, CBAM utilizes attention sequentially in two pivotal dimensions: the channel and the spatial dimensions. First, the channel attention module manages to focus on features and weight each channel, thereby allowing the model to emphasize meaningful features while suppressing irrelevant ones. After applying channel attention, the spatial attention module highlights where the important information resides within the feature map to aid in the model’s concentration on significant portions of space (Woo et al., 2018). Integrating CBAM into the network allows the model to pay more attention to essential feature representations at no considerable cost to computational resources. Therefore, CBAM is beneficial for feature refinement, model interpretability, and performance in classification, detection, and segmentation tasks in computer vision.

## 4 Proposed methodology

This section provides the detailed explanation of the proposed CBA-TransNet model for the classification of OSCC images. This model integrates pre-trained ResNet50 as the feature extractor to extract the local spatial features, followed by a ViT enhanced with the CBAM which is used at the transformer encoder block after the MHSA and FFN layer for the improved attention refinement in global context modeling.

### 4.1 Pre-processing

All the input images were resized to 224 × 224 pixels of uniform resolution, for ensuring the compatibility with the DL architecture. In order to improve the generalization of the model while also reducing its chances of over fitting, certain data augmentation techniques were used, including random horizontal flipping, random rotation not exceeding 15 degrees, and color jittering, which alters brightness, contrast, saturation, and hue. These augmentations excite some of the possible changes that may occur during the acquisition of histopathology images, thereby enhancing the model. Following this stage, the images were converted to normalized tensors using the tensor operator, which normalizes pixel intensity values in the 0 to 1 range.

### 4.2 ResNet50 as feature extractor

**Figure 2:**
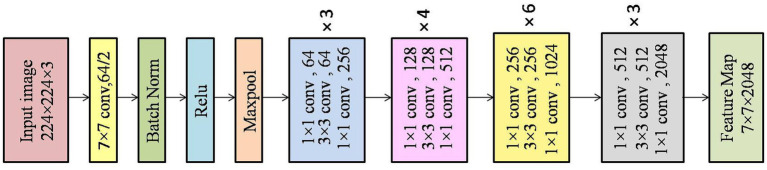
Architecture of ResNet50.

**Figure 3:**
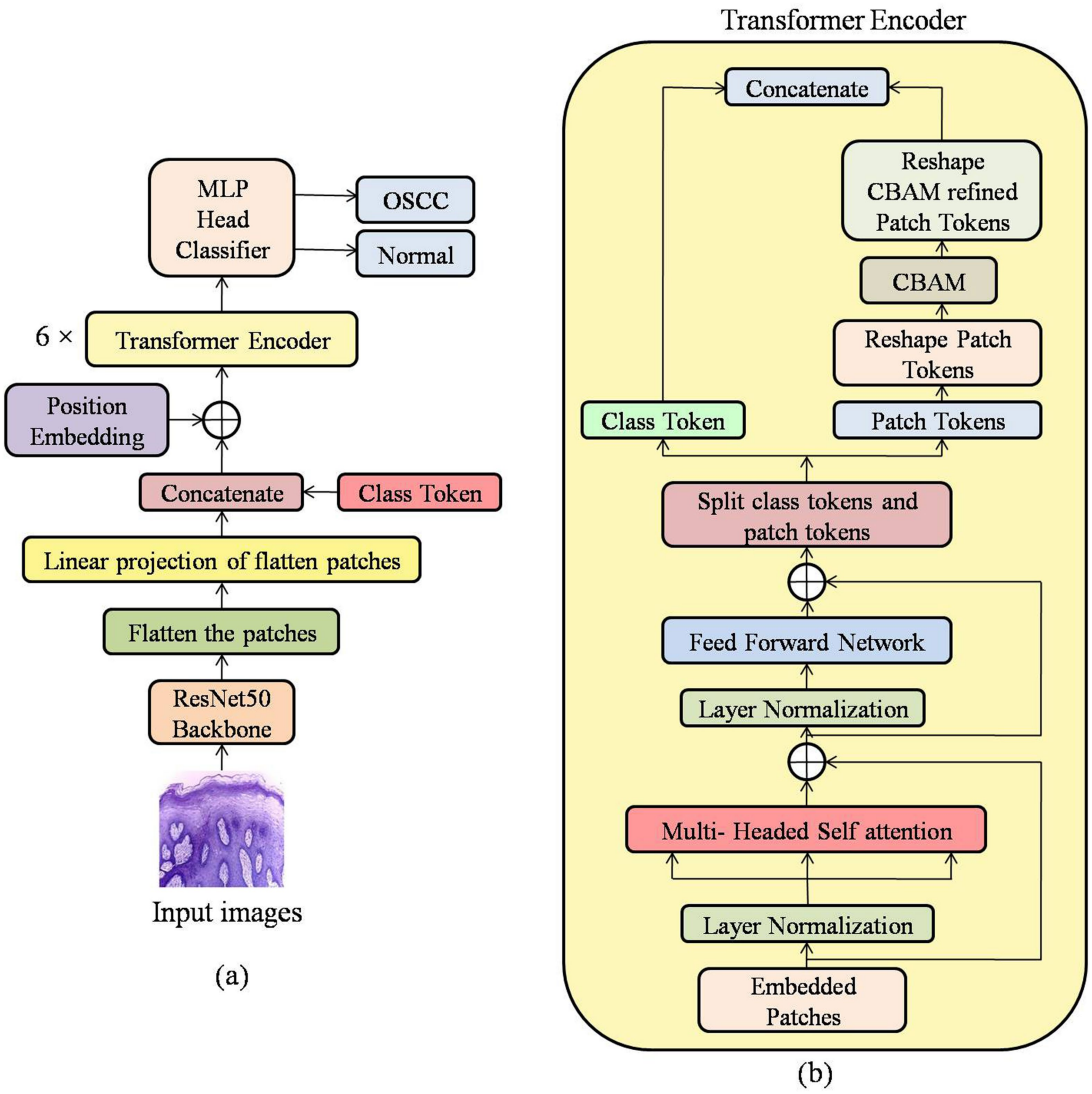
**(a)** Architecture of the proposed CBA-TransNet. **(b)** Schematic of the transformer encoder block in **(a)**.

The input images are initially resized, pre-processed and they are applied to pre-trained ResNet50 network, which is pre-trained on ImageNet dataset to extract features from the image, and it is then processed as a feature map as in Figure 2. The output feature map is then flattened to form patch tokens which can be processed by transformers. In our work the input images of size 224 × 224 × 3 are passed through the pre-trained ResNet50 for a feature extraction where the classification layers are eliminated to get a feature map of size 7 × 7 × 2048. This feature maps are reshaped into 2D tensors and flattened into non overlapping patch tokens, the feature dimension is reduced from 2048 to 512 by applying through linear projection layer. The patch tokens are used together with the class token which marks the beginning of the sequence and these tokens are sent to a ViT encoder layer, the proposed CBA-TransNet model uses 6 encoder layers as shown in Figure 3.

Each of the transformer encoder blocks contains MHSA layers with 8 heads, which extract global dependencies between the patch tokens. The token representations are further modified and enhanced using an FFN. The output from the FFN is splitted into class tokens and patch tokens, in conjunction with this model enhancement, CBAM is used to focus on important clinical features and is applied only on the patch tokens. CBAM performs channel and spatial attention in sequence to adaptively change the focus of the patches to prioritize essential features while ignoring the irrelevant ones and after refinement patch tokens are concatenated with class tokens.

The last step is constructing a classification head, where a dense layer is added to the final output of the ViT encoder to receive the refined patch tokens and the class token. To summarize information and provide a global overview of the entire sequence, class token aggregates the information and is later used for binary classification to distinguish between normal and OSCC images.

### 4.3 Transformer encoder with CBAM

In the proposed CBA-TransNet architecture, each transformer encoder block aims to further refine the representations of tokens utilizing global self-attention, non-linear transformations, and attention augmentation in a sequential manner. The input to the $n^{\mathrm{th}}$ encoder layer is denoted as in Equation 7 𝐵 stands for batch size, *d* for embedding dimensions and 𝑁 for number of patch tokens.


$$X_{n}\epsilon \;R^{B\times (N+1)\times d}$$ (7)

The first operation consists of Layer Normalization (LN) to make the inputs stable, and it is followed by the implementation of MHSA to enable learning of global interdependencies among the tokens as denoted in Equation 8.


$$Y_{n}=X_{n}+\text{MHSA}(\mathrm{LN}(X_{n}))$$ (8)

It captures relationships among all tokens globally. Thus, it allows the model to understand how far apart patches in an image are yet contextually related. Residual addition serves to retain the original input and helps to stabilize training by improving gradient flow. Output received from the attention step is normalized using LN and is then forwarded into a FFN layer as in Equation 9 with linear transformation and Gaussian Error Linear Unit (GELU) activation.


$${\hat{\;Y}}_{n}=Y_{n}+\mathrm{FFN}(\mathrm{LN}(Y_{n}))$$ (9)

The output is divided into class tokens and patch tokens following the FFN layer, as indicated by Equation 10.


$${\hat{\;Y}}_{n}=Y_{\mathrm{cls}};Y_{\mathrm{pat}}$$ (10)

The patch tokens are then processed through the CBAM to refine the appropriate features, as illustrated in Equation 11.


$$Y_{\text{cbam}}=\text{CBAM}\;(Y_{\mathrm{pat}})$$ (11)

As mentioned in Equation 12, CBAM refined patch tokens are again concatenated with the class token, and the resulting output is classified by the MLP head classifier.


$$Y_{\text{output}}=Y_{\mathrm{cls}}+Y_{\text{cbam}}$$ (12)

### 4.4 Advantages and limitations of CBA-TransNet

The proposed CBA-TransNet model improves classification performance by combining ResNet50 for local feature extraction with ViT for global dependencies. To improve channel and spatial attention on patch tokens, CBAM is added after the FFN layer in the transformer encoder block, focusing on clinically significant features. This strategy covers both local and global feature learning, resulting in improved accuracy and interpretability in OSCC classification. However, there are several problems to overcome, such as depending heavily on shallow medical imaging features produced from pre-trained neural networks and converting patch tokens into CNN features, which may result in the loss of some critical minute spatial data. In addition, hyperparameter optimization and robust fine tuning on the CNN-Transformer interaction is needed to gain an optimal model. Inspite of these challenges, the approach has substantial promise toward accurate and interpretable medical image classification.

The proposed method lacks to address the domain-shift problem caused by the heterogeneity of data, imaging devices, and stain methods, which can have an impact on generalization for real-world clinical applications. The diversity of stains in H&E-stained histopathological images, which might result from varying lab preparation and acquisition settings, is another issue that can affect the model’s robustness. Furthermore, the model does not account for the clinically significant variability produced by multiple magnification levels, which is critical for a comprehensive evaluation of the pathology. Future research will address these constraints using techniques like as stain normalization, domain adaptation, and multi-magnification learning.

## 5 Results and discussion

This section outlines a detailed description of the dataset and a brief description of the implementation details used in this work. It also covers hyperparameter optimization alongside the evaluation metrics in which the model was assessed. The detailed summary of the results, including a comparison with the state-of-the-art models.

### 5.1 Dataset description

In this study, we used Histopathological Oral Cancer dataset, which is publicly available online (Histopathological Oral Cancer Dataset, 2021), comprises of 5,192 H&E stained histopathological images balanced with 2,494 normal images which represents 48% of total images and 2,698 OSCC images which represents 52% of total images with a 100× and 400× magnifications.

### 5.2 Implementation details

All the implementations were conducted on a workstation equipped with an Intel Core i5-13600K CPU, 32 GB RAM, and an NVIDIA GeForce RTX 3050 GPU with 8 GB of VRAM. Models were built using Pytorch version2.5.1 alongside CUDA 12.1 in a Jupyter Notebook environment. All the layers of ViT in the CBA-TransNet were trained from scratch on the histopathology oral cancer dataset. Before training a model, all the input images were followed through a standard preprocessing and augmentation pipeline. At first all the images were resized to 224 × 224 pixels. A range of strategies were deployed to prevent model overfitting, thus increasing augmentation efficacy. These strategies comprise of random horizontal flipping, random rotations, and color jittering (controlled brightness, contrast, saturation, and hue shift boundaries). Following this stage, the images were converted to normalized tensors using the tensor operator.

The dataset was constructed for the purpose of model training and evaluation. It is split into training and testing data sets with a split ratio of 70:30. Sample pytorch random split functions were used to provide random yet reproducible sample allocation to partitions. Training data was then divided into a batch size of 32, thereby introducing random order from the model in higher epochs. Test data was also batched with the same size, however, it is retained in a fixed order (not shuffled) to maintain consistent evaluation. A random seed generator and a worker initialization function were introduced into the data loading pipeline across experimental runs to guarantee reproducibility.

### 5.3 Metrics for evaluation of models

The four components of a confusion matrix contents—True Positive (TP), True Negative (TN), False Positive (FP), and False Negative (FN)—are used to assess the effectiveness of the suggested approach. The term TP describes an image that features OSCC and is appropriately identified by the model. TN is an image that is appropriately defined as normal image. FP is the classification of normal image under OSCC, while FN denotes the inaccurate classification of an OSCC image as a normal image. The following formula was used to calculate performance metrics, including accuracy, precision, sensitivity, specificity and F1 score.

Accuracy: It is a metric that quantifies the model’s overall correctness. The analysis can be expressed in terms of the number of correctly classified cases in comparison to the total number of cases.


$$\text{Accuracy}=\frac{\mathrm{TP}+\mathrm{TN}}{\mathrm{TP}+\mathrm{TN}+\mathrm{FP}+\mathrm{FN}}$$ (13)

Precision: It measures the ratio of those who accurately predicted positive occurrences to those who were classified as positive.


$$\text{Precision}=\frac{\mathrm{TP}}{\mathrm{TP}+\mathrm{FP}}$$ (14)

Sensitivity (Recall): It measures the model’s capacity to accurately classify positive occurrences. It shows how effectively the model does not miss out on real positives.


$$\text{Sensitivity}=\frac{\mathrm{TP}}{\mathrm{TP}+\mathrm{FN}}$$ (15)

Specificity: It measures the ability of a model to accurately capture negative occurrences. It shows how well the model mitigates the risk of false positives by accurately identifying negatives.


$$\text{Specificity}=\frac{\mathrm{TN}}{\mathrm{TN}+\mathrm{FP}}$$ (16)

F1 score: It measures both false positives and false negatives, calculated as the harmonic mean of precision and recall.


$$F1\;\text{score}=2\times \frac{\text{Precision}\times \text{Recall}}{\text{Precision}+\text{Recall}}$$ (17)

### 5.4 Optimization of hyperparameters

The proposed CBA-TransNet model has been developed using selected hyperparameters to improve accuracy; the hyperparameter values are fixed prior to training based on preliminary experiments performed on the training data. To support the pre-trained ResNet50 and ViT models, the input image dimensions were resized to 224 × 224. Using 70:30 split ratios, the dataset was split into training and testing sets, with 70% of the data used for training and the remaining 30% for testing. The Adam optimizer set to 0.0001 as the initial learning rate. Cross-Entropy loss was used to model the binary classification of OSCC images as shown in Table 2. Dropout of 0.1 is used after the addition of positional embedding in the patch embedding layer and dropout of 0.2 is used in the FFN layers of the transformer encoders to avoid overfitting.

**Table 2:** Hyperparameters used in proposed CBA-TransNet.

Hyperparameter	Value
Optimizer	Adam
Learning rate	0.0001
Loss Function	Cross entropy
Batch size	32
Epochs	25
Input size	224 × 224 × 3
Transformer encoder layers	6
Number of heads	8

### 5.5 Qualitative CBAM visualization

**Figure 4:**
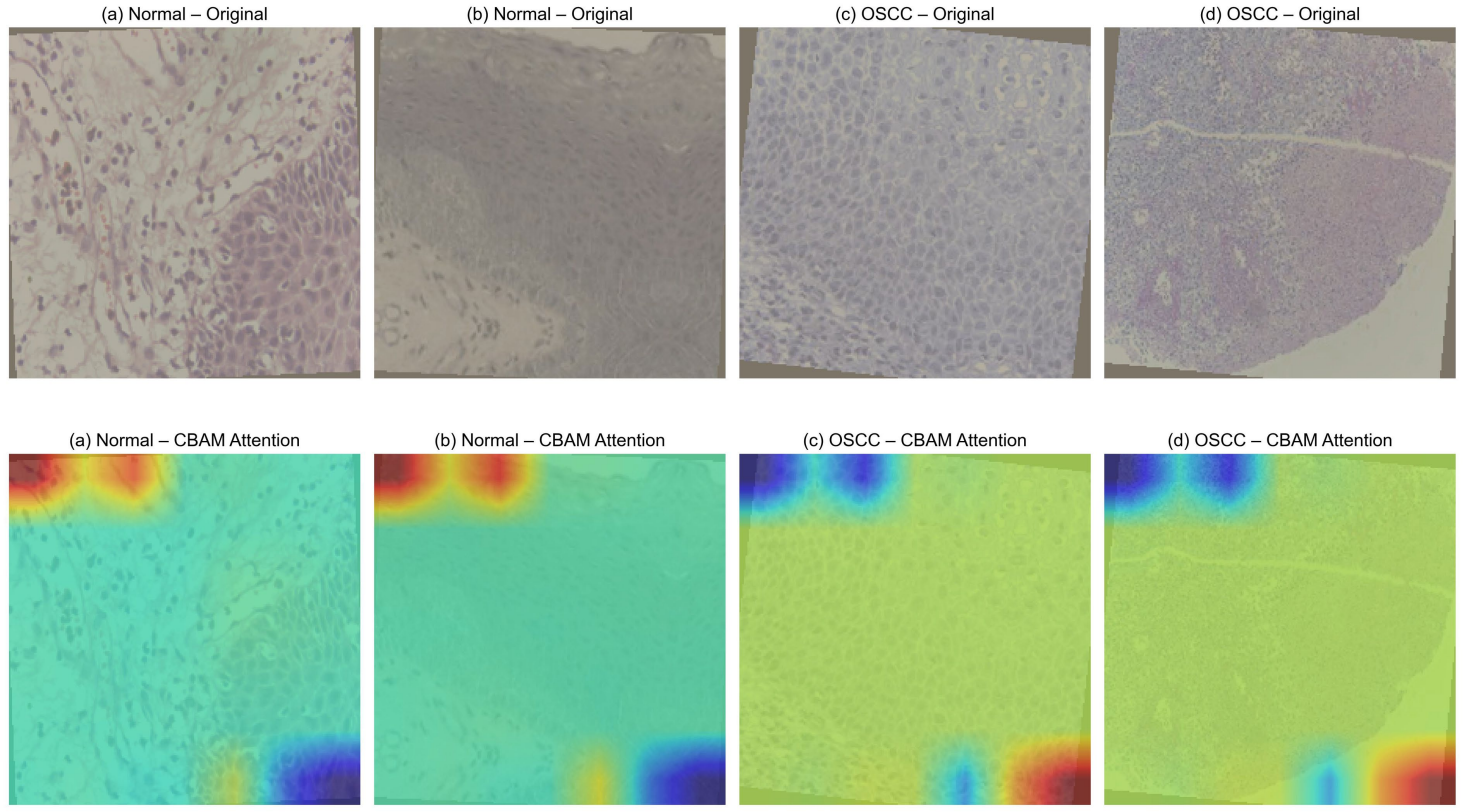
Qualitative CBAM visualization.

The CBAM attention maps were qualitatively evaluated across the normal and OSCC histopathological images to improve the interpretability of the proposed CBA-TransNet model and determine whether predictions were based on diagnostically relevant features as shown in Figure 4, where the top row indicates the original histopathological images and the bottom row indicates the CBAM attention map overlay images. The attention visualization helps the model to focus on spatial regions for model classification. In normal tissue samples, the attention map tends to focus more on the epithelial structures. On the other hand for OSCC samples the attention maps are active in regions characterized by higher cellular density and architectural irregularity tissue morphology. These areas include the clinically important features of pathologically descriptive, irregular epithelial structure, and tumor involvement. The model is activated by the features and is working to eliminate those structures and introduce new malignant features. The attention response is higher near image boundaries or corners. This can be attributed to the coarse spatial resolution of CBAM attention maps, which are generated from downsampled feature representations and then upsampled back to their original size. The attention that the CBAM module provides qualitatively gives evidence that there are histopathological areas of interest are correct and enhances the model.

### 5.6 Performance analysis

The proposed CBA-TransNet was evaluated on the histopathological oral cancer dataset using a 70:30 train–test split ratio with 25 epochs. The evaluation metrics including accuracy, precision, sensitivity, specificity, and F1-score were computed from the confusion matrix using Equations 13–17. On the training set, the model achieved accuracy of 98.38%, precision of 98.92%, sensitivity of 97.92%, specificity of 98.86% and F1-score of 98.42%, while on the testing set, it obtained accuracy of 98.97%, precision of 99.15%, sensitivity of 98.90%, specificity of 99.05%, and F1-score of 99.02%. The train and test results highlight the discriminative capability of CBA-TransNet for OSCC and normal histopathological images classification on the evaluated dataset. Table 3 shows the results of evaluation metrics of the proposed CBA-TransNet model.

**Table 3:** Results of proposed CBA-TransNet.

Split (70:30)	Accuracy (%)	Precision (%)	Sensitivity (%)	Specificity (%)	F1-score (%)
Train	98.38	98.92	97.92	98.86	98.42
Test	98.97	99.15	98.90	99.05	99.02

Table 4 illustrates the 5-fold cross-validation results, which demonstrate the model’s reliability. The model is trained on four folds and tested on one of each fold. The metrics are generated using test data, and the model obtained an average accuracy of 97.50%, specificity of 97.90%, sensitivity of 97.09%, precision of 98.06%, and F1 score of 97.57% indicate that the model has well-distributed classification abilities. The results obtained throughout the test folds suggest that the model has a consistent performance across each fold. Although the 5-fold cross-validation results support the consistency of the model’s performance across varying data partitions, the 70:30 split shows the best overall classification performance on the held-out test set, indicating its use in the final model evaluation.

**Table 4:** 5-fold cross-validation results of proposed CBA-TransNet.

Fold	Accuracy (%)	Precision (%)	Sensitivity (%)	Specificity (%)	F1-score (%)
Fold-1	97.98	98.84	97.15	98.83	97.99
Fold-2	97.59	98.27	96.96	98.25	97.61
Fold-3	97.88	98.57	97.52	98.31	98.04
Fold-4	96.92	97.43	96.29	97.53	96.86
Fold-5	97.11	97.20	97.54	96.58	97.37
Average	97.50	98.06	97.09	97.90	97.57

**Figure 5:**
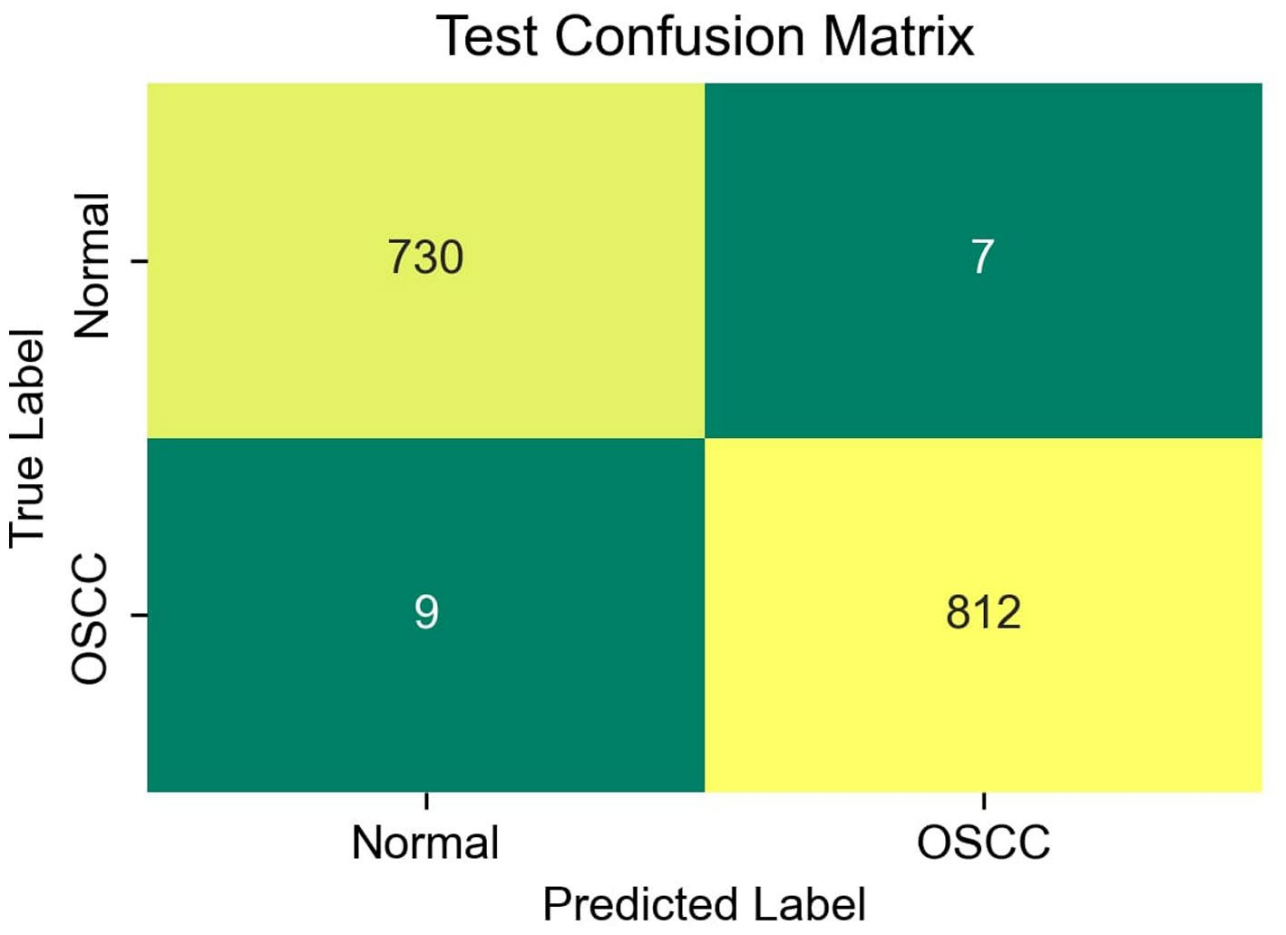
Confusion matrix of proposed CBA-TransNet.

The confusion matrix in Figure 5 confirms the effective classification capability of the CBA-TransNet model. Accuracy is maintained high with 730 samples identified as normal and 769 samples as OSCC. Misclassification rates are also low with 7 false positives and 9 false negatives. This demonstrates a good sensitivity to specificity balance, very crucial to medical image classification where the chances of misdiagnoses must be minimized. The confusion matrix’s diagonal dominance further proves the model’s effective classification on test data and thus reinforces the claim that the proposed CBA-TransNet, which is CBAM refined further improves feature representation and enhances classification results.

**Figure 6:**
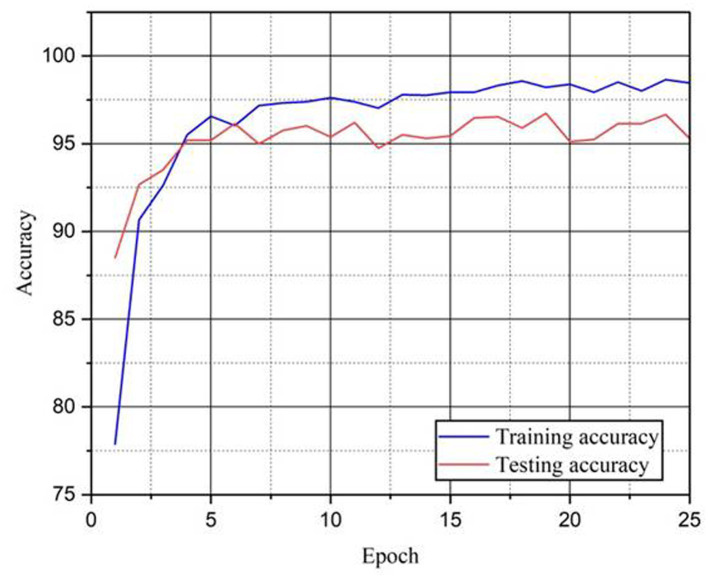
Training and testing accuracy of proposed CBA-TransNet.

**Figure 7:**
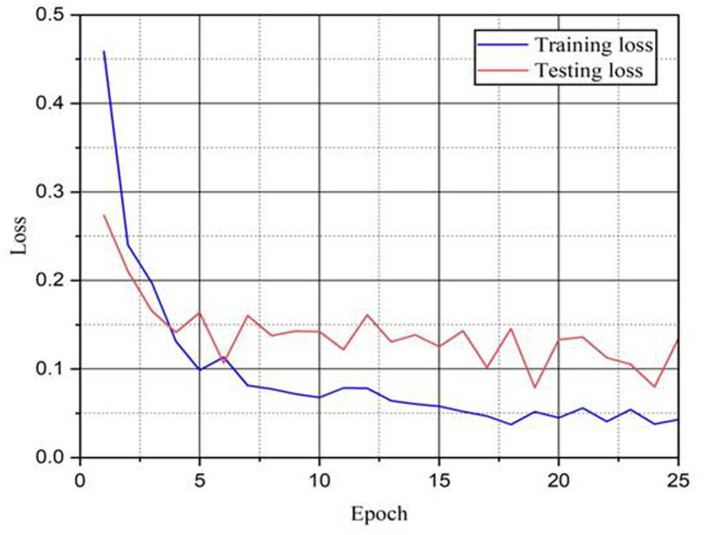
Training and testing loss of proposed CBA-TransNet.

Figure 6 shows the training and the testing curves of the proposed CBA-TransNet model over 25 epochs. Starting with the first epoch, there is a sharp rise in both training and testing accuracy, signaling quick convergence. The training accuracy continues to increase, ultimately surpassing 99% while testing accuracy stabilizes around 95–97% which indicates stable learning capability. Figure 7 shows the training and testing loss curves of the proposed model over 25 epochs and 70:30 train–test split ratios. Both curves show a steep drop within the first few epochs, showing a rapid convergence and optimization of the model. Training loss drops continually to ~0.04, while testing loss stays between 0.08 and 0.16 which shows the stable learning.

### 5.7 Ablation study of proposed CBA-TransNet

Table 5 represents the performance analysis of the ablation study. The study showcases the impacts of CBAM and ResNet50 on the performance of the proposed CBA-TransNet model for the OSCC classification. The proposed CBA-TransNet with the pre-trained ResNet50 as a feature extractor along with CBAM achieved higher accuracy of 98.97% while comparing with the other two variants. Having pre-trained ResNet50 as the backbone in the CBA-TransNet without CBAM results in an accuracy of 98.33% which is slightly lesser compared to the accuracy in the case with CBAM. A similar trend is observed in other metrics. This shows that having convolutional features together with transformer features has an impact on proposed model’s performance. The CBA-TransNet without ResNet50 variant shows moderate performance with 60.53% accuracy which is much lesser compared to the case with ResNet50. This shows the significance of having a hybrid network.

**Table 5:** Ablation study of proposed CBA-TransNet.

Model	Accuracy (%)	Precision (%)	Sensitivity (%)	Specificity (%)	F1-score (%)
**Proposed CBA-TransNet**	**98.97**	**99.15**	**98.90**	**99.05**	**99.02**
CBA-TransNet without CBAM	98.33	98.40	98.40	98.26	98.40
CBA-TransNet without ResNet50	60.53	63.94	52.82	68.64	57.85

Bold values indicate the highest value among the compared methods.

Table 6 shows the results of the ablation studies using 5-fold cross-validation. The proposed CBA-TransNet achieves an accuracy of 97.50%, when the CBAM module is removed the accuracy reduces to 97.28%, demonstrating that it plays a supporting role in feature enhancement. However, removing the ResNet50 backbone results in a significant loss of accuracy down to 65.10% and it demonstrates that ResNet50 drives feature extraction and categorization.

**Table 6:** Ablation study of 5-fold cross-validation results of proposed CBA-TransNet.

Model	Accuracy (%)	Precision (%)	Sensitivity (%)	Specificity (%)	F1-score (%)
**Proposed CBA-TransNet**	**97.50**	**98.06**	**97.09**	**97.90**	**97.57**
CBA-TransNet without CBAM	97.28	96.98	97.84	96.64	97.41
CBA-TransNet without ResNet50	65.10	67.49	63.29	67.04	65.04

Bold values indicate the highest value among the compared methods.

### 5.8 Performance comparison of proposed CBA-TransNet

Table 7 compares the accuracy of the proposed CBA-TransNet model with ViT, CNN, pre-trained ResNet50 models and several deep learning approaches. A traditional CNN model achieved an accuracy of 80.42% while a plain ViT model achieved an accuracy of 69.90%. A pre-trained ResNet50 model achieved an accuracy of 98.59% while the deep learning approaches like AlexNet (Rahman et al., 2022), DenseNet201 + SVM (Ahmad et al., 2023), 10-layered CNN (Das et al., 2023), ViT-14 (Deo et al., 2024b), EfficientNetB0 + DAN (Soni et al., 2024) and Ensemble Classifier (ECOMD) (Das et al., 2024) models achieved an accuracy of 90.06, 97, 97.86, 97.78, 91.1 and 97.88%, respectively. The proposed CBA-TransNet model, on the other hand, achieved an accuracy of 98.97%, which achieved higher accuracy on the evaluated dataset and showing competitive performance in combining convolutional feature extraction, transformer based learning of representations and attention refinement.

**Table 7:** Performance comparison with other DL models and state-of-the-art models.

Model	Accuracy (%)
CNN	80.42
ViT	69.90
Pre-trained ResNet50	98.59
AlexNet (Rahman et al., 2023)	90.06
DenseNet201 + SVM (Ahmad et al., 2023)	97
10-layered CNN (Das et al., 2023)	97.86
ViT-14 (Deo et al., 2024b)	97.78
EfficientNetB0 + DAN (Soni et al., 2024)	91.1
Ensemble Classifier (ECOMD) (Das et al., 2024)	97.88
**Proposed CBA-TransNet**	**98.97**

Bold values indicate the highest value among the compared methods.

Table 8 shows the comparison between the proposed CBA-TransNet and the pre-trained ResNet50 baseline on the Mendeley Histopathological Imaging Database for oral cancer analysis dataset (Mendeley Data, 2022), which has 201 normal images and 495 OSCC images at 400 × magnification with a 70:30 split. The CBA-TransNet achieved 94.74% accuracy and 95.39% sensitivity, and the pre-trained ResNet50 baseline had an accuracy of 92.82% and a sensitivity of 93.88%, indicating that the proposed hybrid model shows promising performance under cross-dataset evaluation. The performance gap between the hybrid CBA-TransNet model and the ResNet50 baseline increased under cross-dataset evaluation, indicating that the transformer-based modules in the hybrid design have the potential to contribute with the changes in image characteristics.

**Table 8:** Comparative analysis on Mendeley Histopathological Imaging Database for oral cancer analysis at 400× magnification.

Approach	Accuracy (%)	Precision (%)	Sensitivity (%)	Specificity (%)	F1-score (%)
Proposed CBA-TransNet	94.74	97.32	95.39	92.98	96.35
Pre-trained ResNet50	92.82	95.83	93.88	90.32	94.85

## 6 Conclusion

The primary aim of this study was to develop a hybrid transformer model for the binary classification of OSCC utilizing histopathological images. The proposed CBA-TransNet model utilizes pre-trained ResNet50 as a feature extractor to gather important high-level local details from the image patches that are later processed by transformer encoders to build the global representation of the input. To improve focus on relevant features, CBAM based attention mechanisms is implemented within transformer encoder blocks after FFN layer by allowing both spatial and channel wise representations to be adaptively refined while considering contention among different parts comprising regions and channels. On a publicly available Histopathological oral cancer dataset consisting of 5,192 histopathological images (Histopathological Oral Cancer Dataset, 2021), the proposed hybrid model resulted in an accuracy of 98.97%, while comparing with the pre-trained ResNet50 baseline (98.59%), ViT (69.90%), CNN (80.42%), AlexNet (90.06%), DenseNet201 + SVM (97%), 10-layered CNN (97.86%), ViT-14 (97.78%), EfficientNetB0 + DAN (91.1%), ECOMD (97.88%). On the Mendeley Histopathological Imaging Database for oral cancer analysis dataset (Mendeley Data, 2022), the proposed model achieved an accuracy of 94.74% compared with the pre-trained ResNet50 baseline of 92.82%, demonstrates that the proposed hybrid model shows promising performance with the changes in image characteristics.

The recommendations for the future work are as follows.

- Extending the classification task from binary to multi-class to include different types and stages of cancer.
- Validating the proposed model with clinically diverse datasets for an adequate measure of the model’s generalizability and real-world clinical applicability.

## Data Availability

Publicly available datasets were analyzed in this study. This data can be found: https://www.kaggle.com/datasets/ashenafifasilkebede/dataset.
